# DGF–YOLOv12n–LiteP2: Dynamic Gated Local–Context Fusion with a High-Resolution Detection Head for Cattle Body-Part Detection in Complex Barns

**DOI:** 10.3390/s26196156

**Published:** 2026-09-28

**Authors:** Yanhong Liu, Le Yang, Qingqing Li, Jiazi Han, Jingrong Wang, Meng Han, Hua Yang

**Affiliations:** 1 College of Information Science and Engineering, Shanxi Agricultural University, Jinzhong 030801, China; yangle3@sxau.edu.cn (L.Y.); hanjiazi@sxau.edu.cn (J.H.); hanm@hdu.edu.cn (M.H.); yanghua@sxau.edu.cn (H.Y.); 2 College of Software, Shanxi Agricultural University, Jinzhong 030801, China; 202530074@stu.sxau.edu.cn (Q.L.); wangjingrong1@sxau.edu.cn (J.W.)

**Keywords:** cattle body-part detection, precision livestock farming, dynamic gated fusion, small-object detection, multi-scale feature aggregation, YOLOv12

## Abstract

Reliable cattle body-part localization provides visual inputs for downstream posture, locomotion, and behavior analysis in precision livestock farming. However, complex barn environments introduce scale variation, occlusion, illumination changes, and rail-like background interference. DGF–YOLOv12n–LiteP2 is presented as a YOLOv12n-based detector that combines dynamic fusion of local and contextual features with a lightweight stride-4 prediction branch. The dynamic gated fusion module integrates multi-kernel local feature extraction, progressive receptive-field aggregation, and input-conditioned channel selection. LiteP2 preserves high-resolution spatial information by reusing the backbone P2 feature without constructing a complete high-resolution feature pyramid. Experiments were conducted on a single-site dataset containing 6991 valid images and 132,302 body-part annotations. On the held-out in-domain test set, DGF–YOLOv12n–LiteP2 achieved 93.92% ${\mathrm{mAP}}_{50}$, 79.27% ${\mathrm{mAP}}_{75}$, and 69.88% ${\mathrm{mAP}}_{50:95}$. Compared with YOLOv12n, ${\mathrm{mAP}}_{50:95}$ increased by 9.61 percentage points. Across three training runs with different random seeds, the mean ${\mathrm{mAP}}_{50:95}$ reached 70.03±0.30%. The model contained 11.68 M parameters and required 22.89 GFLOPs, indicating a trade-off between detection accuracy and computational cost. These results support the complementary roles of dynamic feature fusion and high-resolution prediction under the evaluated barn conditions. Further validation on additional farms, longer acquisition periods, and agricultural edge devices remains necessary.

## 1. Introduction

Precision livestock farming (PLF) converts continuous sensing into timely animal-level information while reducing the labor demand and animal disturbance associated with manual observation [1,2]. Camera-based systems offer non-contact monitoring of multiple animals and support several monitoring tasks through one sensing platform. Nevertheless, illumination and viewpoint variation, frequent occlusion, and complex barn backgrounds continue to constrain reliable cattle monitoring [3,4]. Robust visual perception in operational barns therefore provides an important foundation for practical intelligent livestock management.

Part-level perception provides finer information than whole-animal localization. The body serves as a stable structural reference, while the head provides cues about orientation, feeding, and drinking. The legs relate closely to posture and gait analysis [5,6]. Localization of these regions can support downstream behavior and health analysis. Cattle body-part detection also presents multi-scale and structure-sensitive challenges. Body regions cover large image areas, heads often occupy only a small region, and legs exhibit thin, elongated, and densely distributed structures. Self-occlusion and inter-animal overlap further reduce the visible extent of these body parts. Rails, trough edges, shadows, and repeated floor textures may resemble anatomical boundaries and trigger false responses. Marked scale changes also occur as animals move between near-field and distant regions within a fixed camera view.

Earlier livestock monitoring relied on manual observation and wearable sensors. These approaches remain useful for activity measurement but cannot directly provide dense spatial localization of several anatomical parts in one image. Long-term operation may also be constrained by sensor cost, maintenance requirements, battery life, attachment stability, and animal disturbance. Conventional pipelines based on handcrafted color, texture, or contour descriptors remain sensitive to illumination and background changes. Whole-animal detectors reduce observation effort, but a single cow-level bounding box does not provide the anatomical detail required for head- and leg-related analysis.

These limitations motivated two-stage refinement and one-stage dense prediction in deep object detection [7,8]. For cattle-part perception, FLYOLOv3 introduced filtering for key-part detection [9]. YOLOv5-ASFF reduced scale mismatch through adaptive spatial feature fusion [10], while YOLOv5-EMA strengthened multi-scale responses through efficient attention [11]. Related pose-estimation studies further demonstrate the value of anatomical localization for cattle behavior and posture analysis [5,6]. The existing advances mainly emphasize cross-scale fusion or general feature recalibration rather than explicit selection between local boundary detail and broader contextual evidence within each detection level.

Two coupled gaps remain. First, local detail and context play image-dependent roles. Fine boundaries aid the detection of small heads and thin legs. However, strong local enhancement may amplify rails and floor textures. Context can suppress these distractors and reduce the effects of occlusion. Excessive aggregation may weaken subtle boundaries. Fixed addition or concatenation cannot balance local and contextual information across images and channels. Second, conventional P3–P5 detection starts at stride 8. Repeated downsampling may remove spatial information before small or slender parts reach the prediction layers. A complete high-resolution feature pyramid can preserve this information. However, considerable memory and computation are required.

To address these gaps, DGF–YOLOv12n–LiteP2 combines dynamic gated fusion with a lightweight high-resolution prediction branch. Dynamic gated fusion (DGF) contains a multi-scale dense convolution block (MDCB) for local-detail extraction. Multi-receptive-field feature aggregation (MRFA) constructs contextual representations. Local and contextual states are then combined by a triplet-conditioned channel gate. Fixed fusion weights are therefore avoided. A zero-initialized bounded spatial correction operates only at P4. Ambiguous responses can be refined while the stable base path remains unchanged.

LiteP2 complements DGF by extending prediction from P3–P5 to P2–P5. The branch reuses the stride-4 backbone feature. One compact high-resolution refinement block precedes bottom-up reconstruction. This design improves spatial representation for small and thin body parts. A heavy full-resolution neck is not required.

### 1.1. Related Works

In this section, we review recent studies related to cattle body-part detection, including modern object detectors, multi-scale feature representation, and adaptive feature fusion methods.

#### 1.1.1. Detection and Multi-Scale Representation

Modern object detectors follow two-stage and one-stage designs. Faster R-CNN refines region proposals before classification and localization [7]. SSD and the original YOLO perform dense prediction in one forward pass [8,12]. Later YOLO families improved optimization, feature reuse, and real-time accuracy. Representative models include YOLOv3 [13], YOLOv4 [14], YOLOX [15], YOLOv6 [16], YOLOv7 [17], and the attention-based YOLOv12 baseline adopted in this study [18].

Feature pyramids support detection across different object sizes. FPN introduced top-down semantic propagation through lateral connections [19]. PANet added bottom-up aggregation [20], while EfficientDet applied a weighted bidirectional pyramid [21]. FCOS demonstrated anchor-free dense prediction across pyramid levels [22]. These designs motivate multi-level detection. However, pyramid topology alone cannot determine whether each channel should emphasize local boundaries or broader context.

Transformer detectors provide another development path. DETR introduced set-based end-to-end prediction [23]. Deformable DETR improved multi-scale convergence through sparse sampling [24]. DINO strengthened denoising and query initialization [25], while RT-DETR targeted real-time end-to-end detection [26]. The experimental comparison therefore includes convolutional and transformer-based references.

#### 1.1.2. Attention and Adaptive Feature Fusion

The transformer architecture established self-attention as a general feature interaction mechanism [27]. Efficient convolutional attention often relies on compact descriptors. Squeeze-and-Excitation recalibrates channels through global pooling [28]. CBAM combines channel and spatial attention [29]. Selective Kernel Networks learn branch selection across receptive fields [30], while coordinate attention embeds directional position information [31]. DGF selects between two feature sources rather than reweighting one tensor. Its gate mixes local and contextual tensors from a descriptor that also includes the unmodified input. This formulation creates an explicit competition between information sources.

Recent studies have also addressed challenging detection conditions from different perspectives. AMIEOD introduces adaptive multi-expert image enhancement for object detection in low-illumination scenes, improving visual information under adverse lighting conditions [32]. DeCenter employs density-center-guided perception enhancement for UAV object detection, with particular attention to small, dense, and occluded targets [33]. These studies demonstrate the importance of condition-aware enhancement and target-sensitive feature representation under challenging imaging conditions. In contrast, cattle body-part detection in complex barns requires the joint consideration of local structural details, contextual background interference, and high-resolution spatial information. The present study therefore focuses on dynamic local–context feature fusion and lightweight high-resolution prediction for body, head, and leg detection.

### 1.2. Research Gap and Study Objective

Prior cattle part detectors demonstrate the value of filtering, cross-scale fusion, and attention [9,10]. Direct numerical ranking across these studies remains inappropriate because datasets, splits, pretraining, and metric implementations differ. A remaining need concerns within-scale fusion between high-frequency part boundaries and long-range contextual constraints. The high-resolution detection head also requires separate evaluation from the fusion mechanism. This study addresses both points through code-level ablations and a paired design.

Accordingly, this study aimed to develop and evaluate DGF–YOLOv12n–LiteP2 for cattle body-part localization under complex barn conditions. Further evaluation examined whether dynamic local–context fusion and a compact stride-4 prediction branch could improve the localization of small and slender body parts while quantifying the associated accuracy–complexity trade-off.

## 2. Materials and Methods

### 2.1. Dataset

The cattle body-part detection dataset was collected from fixed surveillance cameras installed at the Jinnan cattle genetic resource conservation center in Yuncheng, Shanxi Province, China. According to the embedded timestamp information, the images were mainly collected from October to November 2023. The monitoring scenes covered indoor corridors, outdoor activity areas, feeding zones, and fenced regions. The dataset contains diverse environmental conditions, including illumination variation, shadows, rail occlusion, background interference, distant targets, crowded cattle groups, and truncated objects. In this study, “complex barns” refers operationally to this combination of heterogeneous monitoring zones and visually challenging conditions rather than a specific standardized housing classification.

The retained records preserve image resolution and embedded timestamps, but the surveillance-camera manufacturer/model, detailed camera installation parameters, and verified animal-level demographic metadata were not available and therefore could not be reported reliably. Images were obtained from routine surveillance rather than predefined time-of-day subsets, and nighttime and low-illumination scenes were included in the qualitative evaluation.

**Figure 1:**
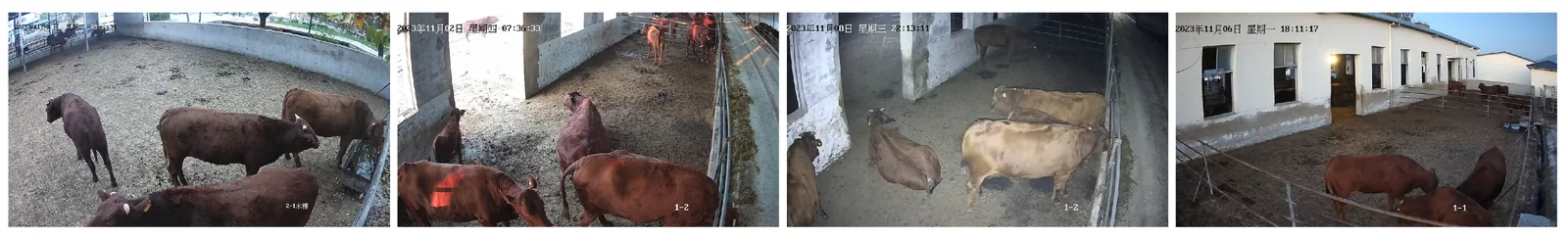
Representative annotations from the self-built dataset. The original Chinese overlays indicate the recording date, weekday, and camera location (e.g., “water trough”); these are surveillance metadata, not annotation labels.

A total of 6994 RGB images were collected, including 6991 valid images after quality control. The image resolutions include 2560×1440 and 1920×1080 pixels. All the images were annotated in COCO format with three categories: body, head, and leg. The dataset was divided into training, validation, and test sets containing 4894, 1398, and 699 valid images, respectively. The final dataset contains 132,302 annotated instances, including 46,432 body instances, 31,301 head instances, and 54,569 leg instances. The dataset statistics are summarized in Table 1, and representative annotations are shown in Figure 1.

### 2.2. Implementation

All the experiments were conducted on a single NVIDIA GeForce RTX 4090 GPU (NVIDIA Corporation, Santa Clara, CA, USA). The software environment comprised Python 3.10.8 (Python Software Foundation, Wilmington, DE, USA), PyTorch 2.1.2 (PyTorch Foundation, hosted by the Linux Foundation, San Francisco, CA, USA), CUDA 11.8 (NVIDIA Corporation, Santa Clara, CA, USA), and Ultralytics 8.3.63 (Ultralytics, Frederick, MD, USA). The input images were resized to 640×640, and the models were trained for 100 epochs with a batch size of 32. AdamW was employed with an initial learning rate of 0.001, a final learning-rate ratio of 0.01, and a weight decay of 0.0005. A cosine learning-rate schedule and a three-epoch warm-up strategy were adopted during training.

### 2.3. DGF–YOLOv12n–LiteP2 Network Architecture

DGF–YOLOv12n–LiteP2 builds on YOLOv12n for cattle body-part detection in complex barn environments. The backbone generates P2, P3, P4, and P5 features at strides of 4, 8, 16, and 32, respectively. DGFBlock modules are placed at the outputs of the P3, P4, and P5 stages. Through dynamic fusion of local details and contextual representations, these modules enhance the multi-scale features entering the neck.

The proposed components target different but complementary challenges in complex barn environments. MDCB uses multi-kernel local feature extraction to preserve fine boundaries and structural details, which benefits the representation of small heads, thin legs, and partially visible body parts. MRFA progressively enlarges the receptive field and introduces broader contextual information, helping to distinguish anatomical structures from rails and repetitive background patterns under occlusion and background interference. The dynamic gating mechanism adaptively balances local and contextual information according to the input features, reducing the risk that excessive local enhancement amplifies background interference. LiteP2 further reuses the high-resolution backbone P2 feature for stride-4 prediction, retaining more spatial information for small and thin body parts under scale variation. These components therefore provide complementary local-detail preservation, contextual discrimination, adaptive feature selection, and high-resolution prediction.

The neck first follows a top-down feature propagation path. After DGFBlock enhancement, the P5 feature undergoes upsampling and channel-wise concatenation with the enhanced lateral P4 feature. The fused feature then passes through separate MRFA and A2C2f modules. Next, the resulting P4 fusion feature undergoes further upsampling and concatenation with the lateral P3 feature. The same processing sequence completes contextual enhancement and feature aggregation at the P3 scale.

LiteP2 further upsamples the P3 fusion feature from the top-down path to a stride-4 spatial resolution. The upsampled feature is then concatenated with the lateral P2 feature from the backbone. The merged feature passes through a C3k2 module to form a high-resolution P2 representation. This branch directly reuses the backbone P2 feature and adds only a compact high-resolution fusion path. The design avoids constructing a complete high-resolution feature pyramid.

**Figure 2:**
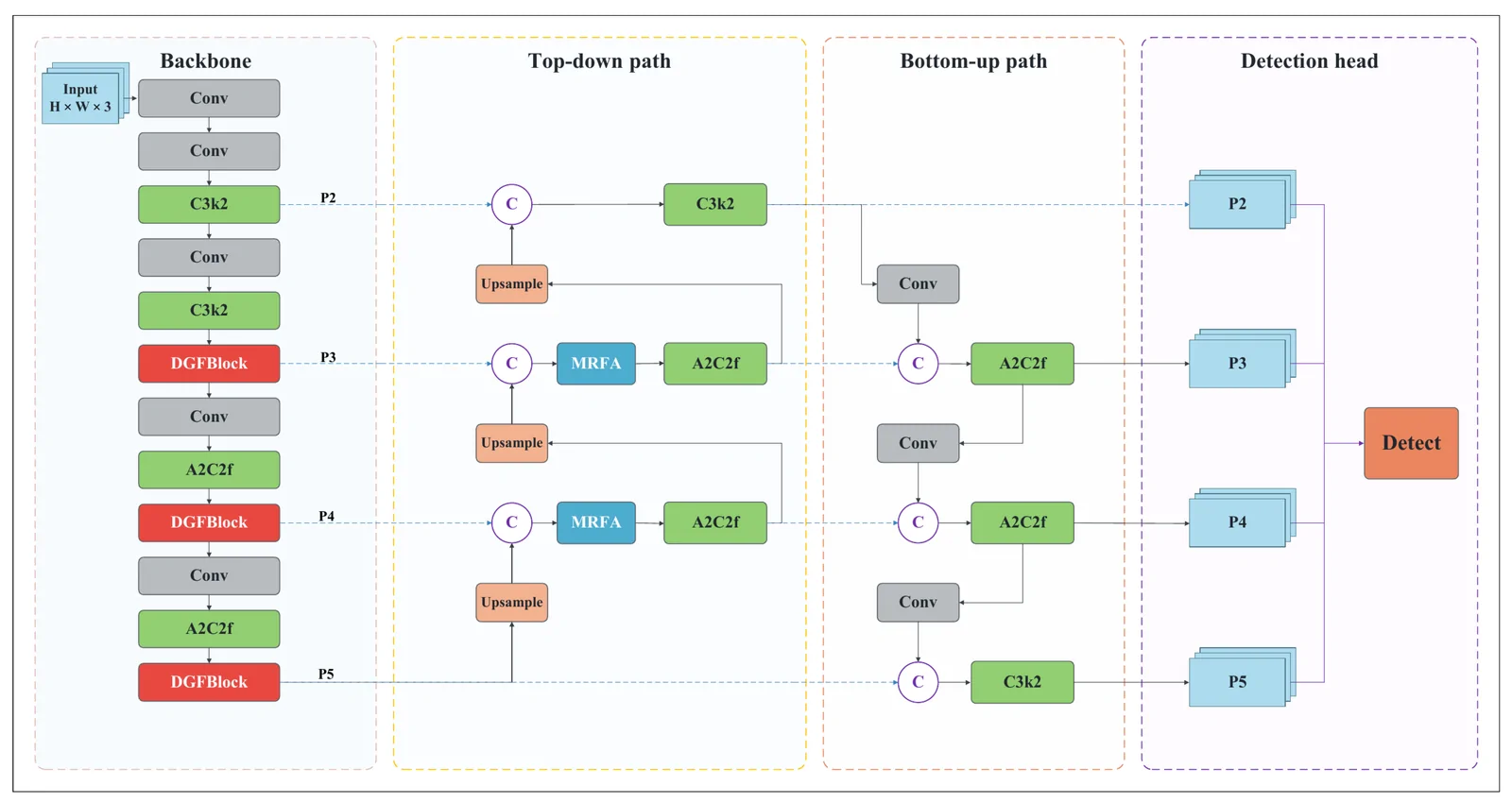
Overall architecture of the DGF–YOLOv12n–LiteP2 network.

The bottom-up path then transfers spatial details from P2 toward deeper levels through strided convolution, feature concatenation, and feature aggregation. This process produces the P3, P4, and P5 fusion features for detection. Finally, the detection head receives the P2 feature from the top-down path and the P3, P4, and P5 features from the bottom-up path. Joint prediction is performed across four feature scales. The overall network architecture is presented in Figure 2.

### 2.4. MDCB Local Detail Extraction

**Figure 3:**
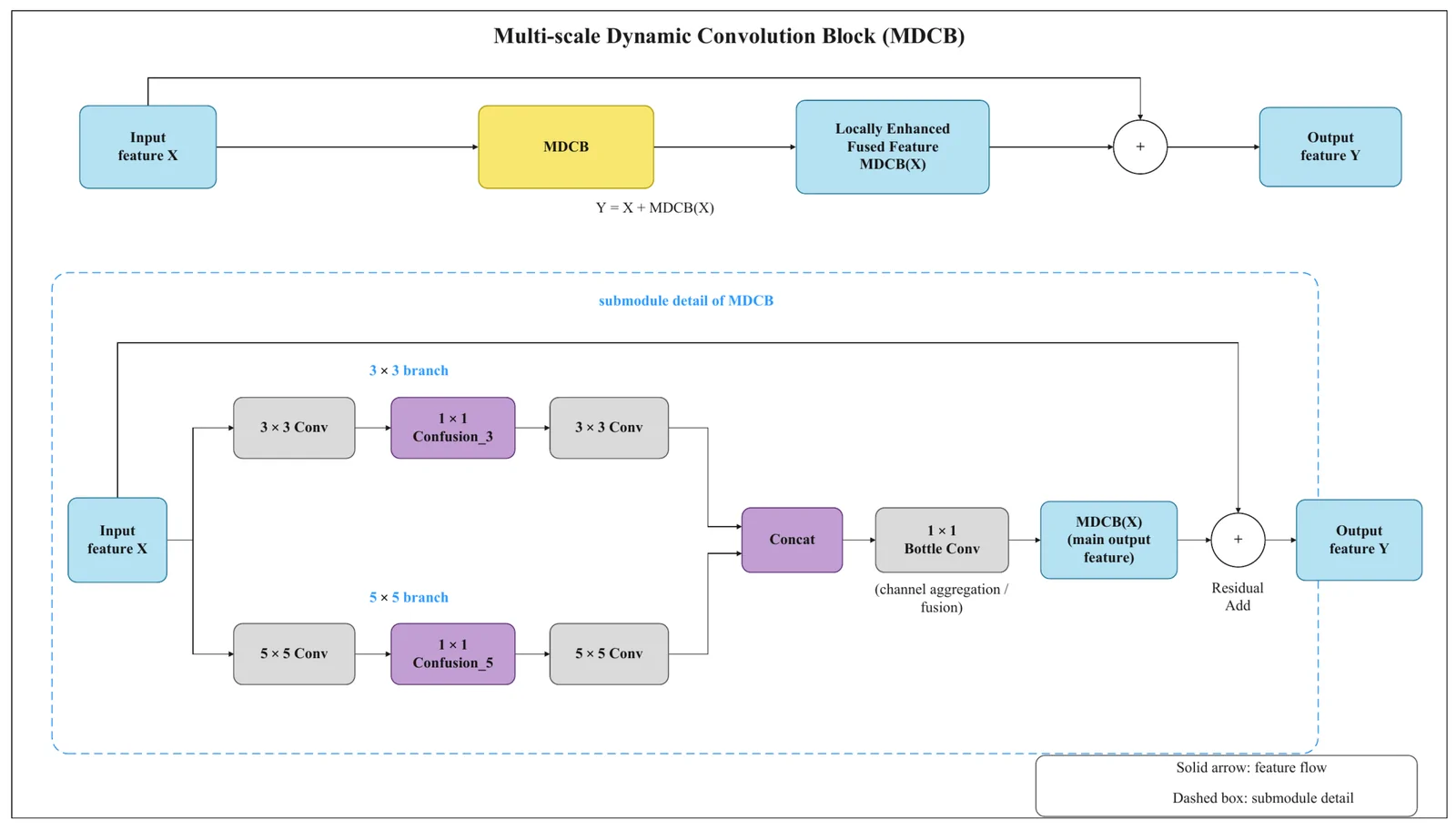
Detailed structure of the MDCB branch.

MDCB extracts structural information under different local receptive fields, as shown in Figure 3. Given an input feature $X\in R^{C\times H\times W}$, the module constructs parallel 3×3 and 5×5 convolution branches. Fine-grained boundaries and local textures are captured by the 3×3 branch. Structural patterns within a broader local region are extracted by the 5×5 branch. Each branch contains two spatial convolutions, with a 1×1 channel mapping inserted between them. For the 3×3 branch, the output takes the following form:

(1) $$U_{3}=\phi_{3}^{2}\left(\kappa_{3}\left(\phi_{3}^{1}\left(X\right)\right)\right),$$

where $\phi_{3}^{1}\left(\cdot \right)$ and $\phi_{3}^{2}\left(\cdot \right)$ denote the first and second 3×3 convolution operations, respectively. The intermediate 1×1 channel mapping is represented by $\kappa_{3}\left(\cdot \right)$.

Likewise, the 5×5 branch produces

(2) $$U_{5}=\phi_{5}^{2}\left(\kappa_{5}\left(\phi_{5}^{1}\left(X\right)\right)\right),$$

where $\phi_{5}^{1}\left(\cdot \right)$ and $\phi_{5}^{2}\left(\cdot \right)$ denote the first and second 5×5 convolution operations, respectively. The corresponding 1×1 channel mapping is denoted by $\kappa_{5}\left(\cdot \right)$.

The outputs of both branches are then concatenated along the channel dimension. A 1×1 projection performs channel fusion and dimensional alignment. The final local feature follows a residual addition,

(3) $$L=X+\rho \left([U_{3};U_{5}]\right),$$

where [·] denotes channel concatenation, and ρ(·) represents the 1×1 projection. The two parallel branches provide complementary local features. The residual path preserves the input representation and supports stable feature propagation.

### 2.5. MRFA Context Aggregation

**Figure 4:**
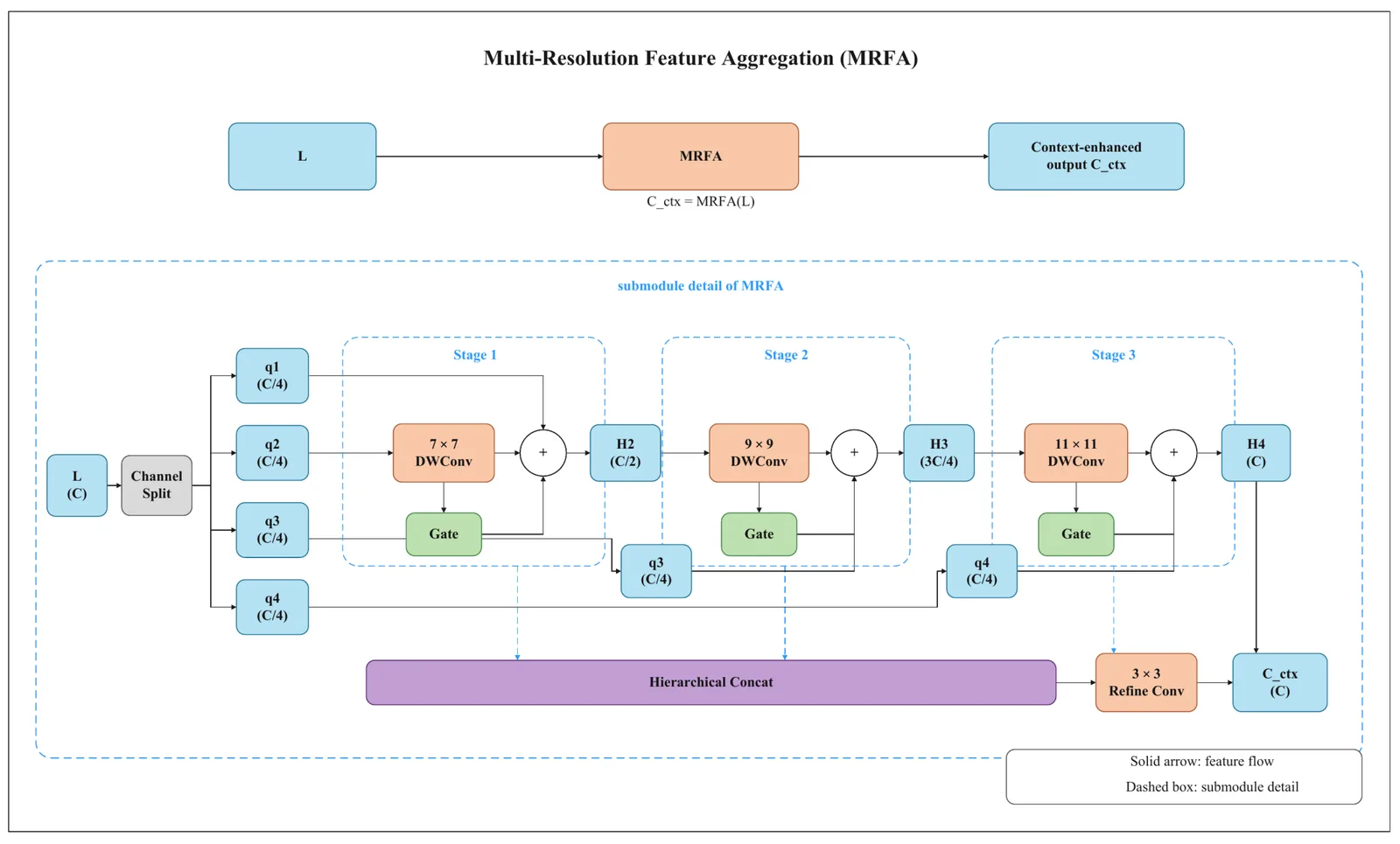
Detailed structure of the MRFA branch.

MRFA extracts contextual information over larger receptive fields, as shown in Figure 4. Layer normalization is first applied to *L* to stabilize the feature distribution:

(4) $$\bar{L}=\mathrm{LN}\left(L\right).$$

Here, $L\in R^{C\times H\times W}$. The normalized feature $\bar{L}$ is evenly divided into four groups along the channel dimension. The partitioned feature is denoted by *Q*:

(5) $$Q={\mathrm{Split}}_{c}\left(\bar{L}\right)=\left[q_{1},q_{2},\ldots ,q_{i}\right].$$

In this expression, ${\mathrm{Split}}_{c}\left(\cdot \right)$ denotes equal partitioning along the channel dimension, and the subscript *c* indicates a channel-wise operation. Each group satisfies $q_{i}\in R^{\frac{C}{4}\times H\times W}$, where *i* denotes the group index and I=4 denotes the total number of groups. Thus, *Q* contains $q_{1}$, $q_{2}$, $q_{3}$, and $q_{4}$. The notation [,] denotes channel concatenation.

The channel groups in *Q* then pass through a three-stage progressive context aggregation process. At the first stage, $q_{1}$ and $q_{2}$ are aggregated to generate $H_{2}$. The second stage incorporates $q_{3}$ into the preceding representation to produce $H_{3}$. The third stage further introduces $q_{4}$ and generates $H_{4}$. As illustrated in Figure 4, the three stages contain 7×7, 9×9, and 11×11 depthwise convolutions, respectively. This progressive design gradually incorporates additional channel groups and expands the receptive field.

Outputs from the three stages are concatenated hierarchically to form the aggregated feature *Z*:

(6) $$Z=\left[H_{2},H_{3},H_{4}\right]=P\left(Q\right).$$

Here, P(·) represents the complete three-stage progressive context aggregation process described above. Specifically, $H_{2}$ contains information aggregated from the first two channel groups, $H_{3}$ extends this representation with the third group, and $H_{4}$ further incorporates the fourth group. Their channel dimensions correspond to $\frac{C}{2}$, $\frac{3C}{4}$, and *C*, respectively. After hierarchical concatenation, the resulting feature satisfies $Z\in R^{\frac{9C}{4}\times H\times W}$.

The hierarchically aggregated feature *Z* then enters a 3×3 refinement convolution. This operation performs channel integration and produces the final context-enhanced feature:

(7) $$C_{\mathrm{ctx}}=R_{3}\left(Z\right).$$

In this equation, $R_{3}\left(\cdot \right)$ denotes a 3×3 refinement convolution with *C* output channels. The resulting feature satisfies $C_{\mathrm{ctx}}\in R^{C\times H\times W}$. The progressive 7×7, 9×9, and 11×11 depthwise convolutions gradually enlarge the receptive field. This design avoids applying large-kernel convolution directly to all the input channels.

### 2.6. Dynamic Gated Feature Fusion

**Figure 5:**
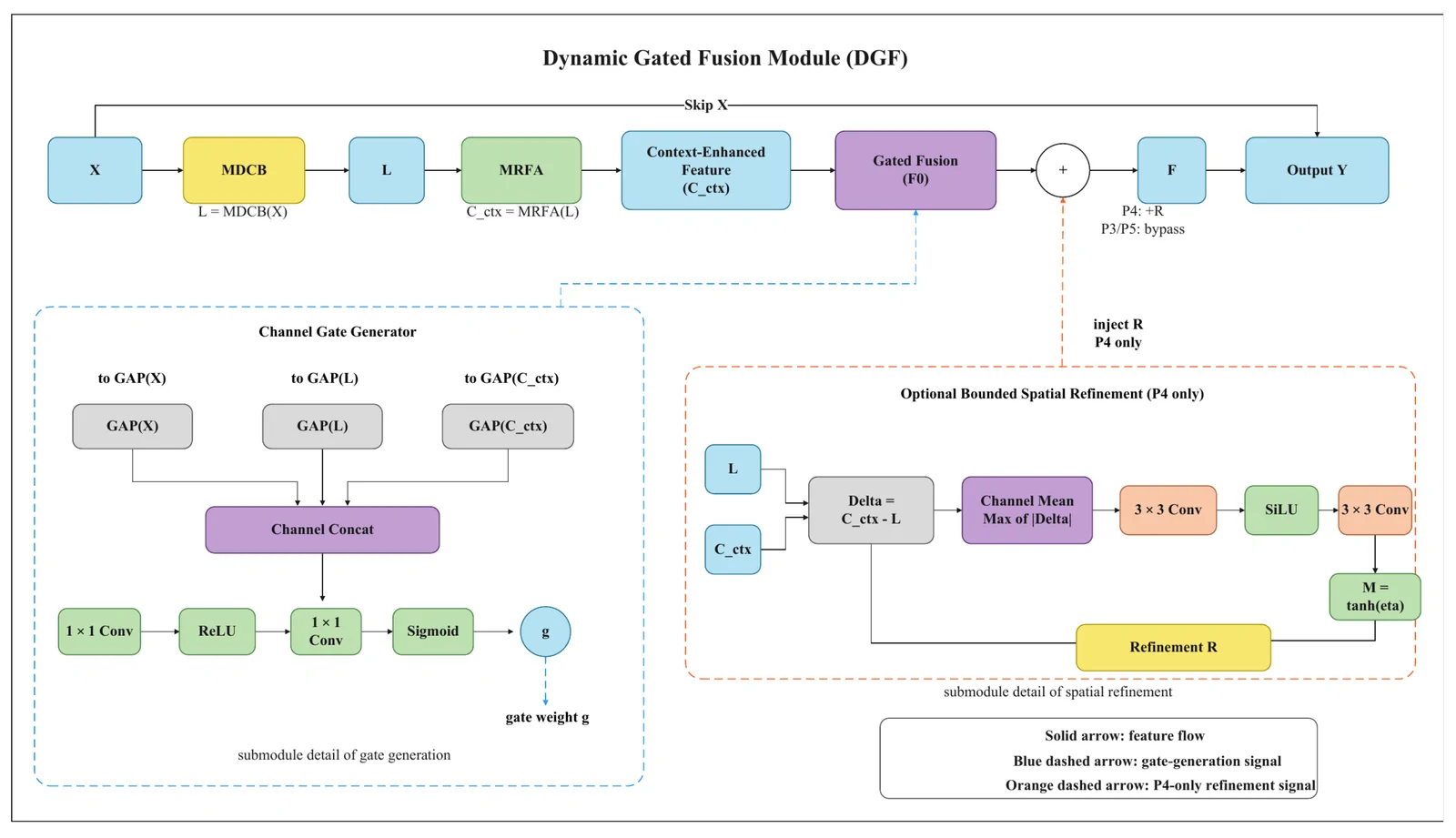
Detailed structure of the DGFBlock branch.

DGF adaptively balances local-detail information and contextual information according to the input content, as shown in Figure 5. Given an input feature $X\in R^{C\times H\times W}$, MDCB first extracts the locally enhanced feature *L*:

(8) L=MDCB(X).

Here, *X* denotes the input feature of the DGF module, while MDCB(·) represents the multi-scale dynamic convolution block. The feature *L* then enters MRFA to obtain the context-enhanced feature $C_{\mathrm{ctx}}$ with a broader receptive field

(9) $$C_{\mathrm{ctx}}=\mathrm{MRFA}\left(L\right).$$

where MRFA(·) denotes the multi-resolution feature aggregation module. Local and contextual features are then combined as follows:

(10) $$F_{0}=g⊙L+\left(1-g\right)⊙C_{\mathrm{ctx}}.$$

Here, $F_{0}$ denotes the initial gated fusion feature, *g* represents the channel-wise gating weight, and ⊙ indicates element-wise multiplication. A larger *g* places greater emphasis on local details, whereas a smaller *g* assigns more weight to contextual information.

The final output feature of DGF is defined as:

(11) $$Y=X+\gamma \left(F_{0}+I_{P4}R-X\right).$$

Here, *X* denotes the original input feature, $F_{0}$ represents the gated fusion feature from the previous stage, and *R* denotes the bounded spatial correction derived from the discrepancy between *L* and $C_{\mathrm{ctx}}$. The indicator $I_{P4}$ takes a value of 1 at the P4 stage and 0 at the P3 and P5 stages. The learnable residual interpolation coefficient γ controls the contribution of the fused feature to the final output.

### 2.7. LiteP2 Prediction Head

The baseline predicts at strides 8, 16, and 32. LiteP2 upsamples P3^*td*^ to stride 4 and concatenates it with the backbone P2 tensor. A single C3k2 block processes this high-resolution feature. The design retains a stride-4 prediction grid with limited branch depth. A later stride-2 convolution returns P2^*out*^ to P3 resolution and starts the bottom-up path. High-resolution cues can therefore influence direct P2 prediction and the reconstructed coarser levels.

### 2.8. Evaluation Metrics

Five standard object detection metrics were adopted to evaluate the performance of different detectors, including precision (P), recall (R), ${\mathrm{mAP}}_{50}$, ${\mathrm{mAP}}_{75}$, and COCO-style ${\mathrm{mAP}}_{50:95}$.

Precision represents the proportion of correctly predicted objects among all the predicted objects,

(12) $$P=\frac{TP}{TP+FP},$$

where TP and FP denote true positive and false positive predictions, respectively.

Recall measures the proportion of correctly detected objects among all the ground-truth objects,

(13) $$R=\frac{TP}{TP+FN},$$

where FN denotes false negative predictions.

Average precision (AP) integrates the precision–recall curve under a specific IoU threshold. The mean average precision (mAP) is calculated by averaging AP values over all the categories:

(14) $$mAP=\frac{1}{C}\sum_{i=1}^{C}AP_{i},$$

where *C* represents the number of object categories.

Following the COCO evaluation protocol, this study reports ${\mathrm{mAP}}_{50}$, ${\mathrm{mAP}}_{75}$, and COCO-style ${\mathrm{mAP}}_{50:95}$. Specifically, ${\mathrm{mAP}}_{50}$ and ${\mathrm{mAP}}_{75}$ denote mAP values at IoU thresholds of 0.50 and 0.75, respectively. The ${\mathrm{mAP}}_{50:95}$ metric averages AP values from IoU 0.50 to 0.95 with an interval of 0.05, providing a comprehensive evaluation of localization accuracy.

For the three-seed evaluations, each metric was summarized by its arithmetic mean and sample standard deviation across the deterministic seeds 0, 1, and 2. Two-sided 95% confidence intervals for the mean were calculated as $\bar{x}\pm t_{0.975,\;2}s/\sqrt{3}$, where *s* is the sample standard deviation and $t_{0.975,\;2}=4.303$. For paired model comparisons, the metric difference was first calculated within each seed and the mean and sample standard deviation of the three paired differences were then reported. No separate null-hypothesis significance test was performed because only three deterministic training runs were available.

The training objective of YOLO-based detectors combines box regression loss, classification loss, and distribution focal loss. The focal loss mechanism follows the formulation introduced in [34], while the distribution focal loss is derived from generalized focal loss.

## 3. Results

### 3.1. Comparative Experiments and Performance Analysis of Different Detection Models

To evaluate the proposed DGF–YOLOv12n–LiteP2, comparative experiments and performance analysis were conducted in this section. Faster R-CNN R50-FPN, DINO R50, RT-DETR-l, YOLOv5n, YOLOv8n, YOLO11n, YOLO11s, and YOLOv12n were selected for comparison. DGF–YOLO and YOLOv12n–LiteP2 were retained as two architecture-matched models. All the comparative results are presented below.

As shown in Table 2, DGF–YOLOv12n–LiteP2 reached 93.71% ${\mathrm{mAP}}_{50}$, 80.11% ${\mathrm{mAP}}_{75}$, and 69.75% ${\mathrm{mAP}}_{50:95}$. These results indicate that the proposed model achieved the highest detection accuracy among the evaluated models on the retained validation set. Relative to YOLOv12n, ${\mathrm{mAP}}_{50:95}$ increased by 9.44 percentage points. The margins over YOLO11s and RT-DETR-l were 0.11 and 0.89 percentage points, respectively.

Table 3 presents the held-out in-domain test results. DGF–YOLOv12n–LiteP2 achieved 91.47% precision, 86.73% recall, 93.92% ${\mathrm{mAP}}_{50}$, 79.27% ${\mathrm{mAP}}_{75}$, and 69.88% ${\mathrm{mAP}}_{50:95}$. Compared with YOLOv12n, DGF–YOLO, and YOLOv12n–LiteP2, the ${\mathrm{mAP}}_{50:95}$ gains were 9.61, 2.83, and 3.80 percentage points, respectively.

To further assess stability, YOLOv12n, DGF–YOLO, YOLOv12n–LiteP2, and DGF–YOLOv12n–LiteP2 were trained from scratch. Three training runs with random seeds 0, 1, and 2 were performed for each model.

According to Table 4, the complete model reached a mean test ${\mathrm{mAP}}_{50:95}$ of 70.03±0.30%. The seed-paired mean gains over YOLOv12n, DGF–YOLO, and YOLOv12n–LiteP2 were 10.76±0.49, 3.68±0.47, and 4.91±0.58 percentage points. All the corresponding 95% confidence intervals remained above zero. The observed improvement therefore remained consistent across the three deterministic random seeds.

**Figure 6:**
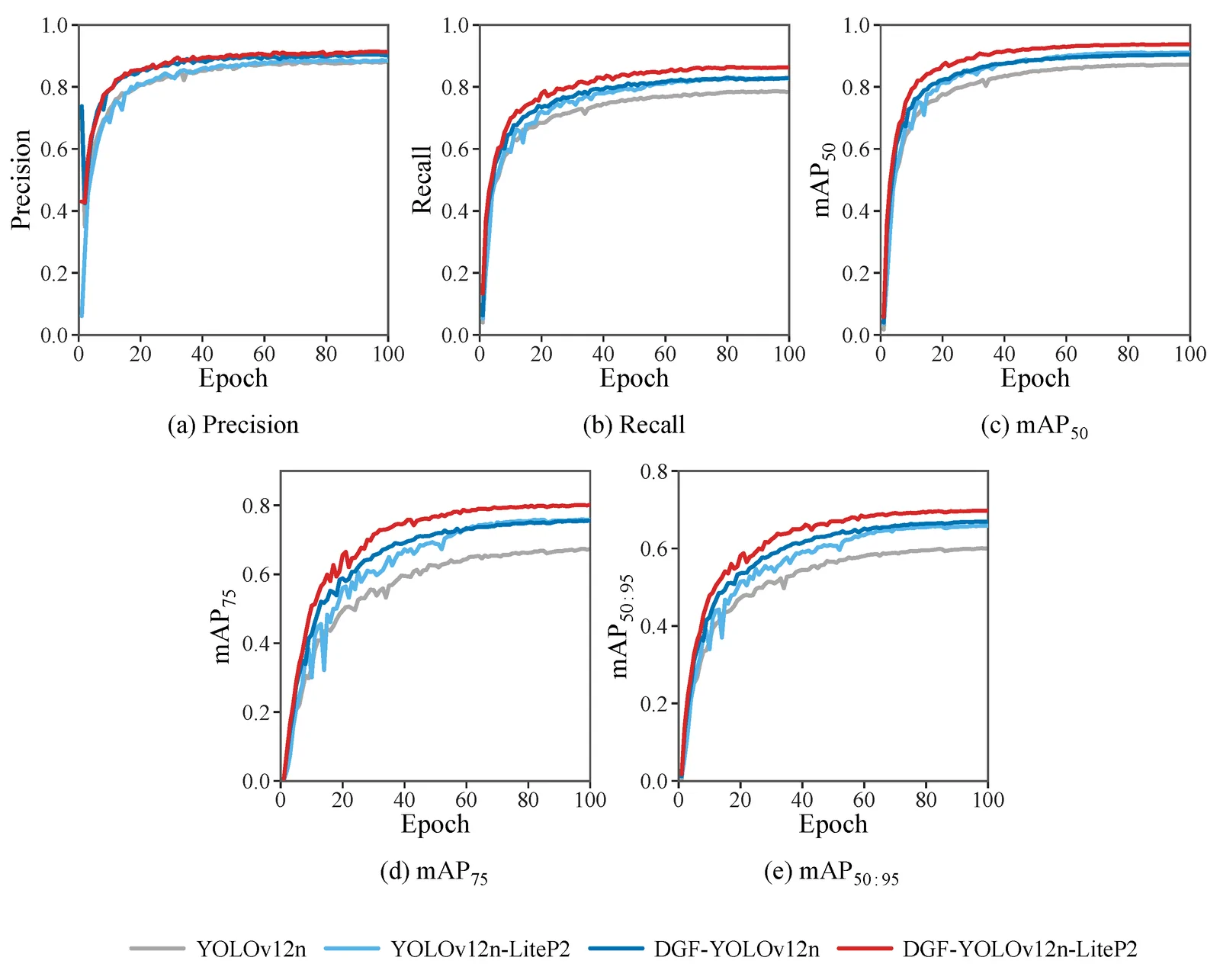
Validation metric curves of four architecture-matched models over 100 training epochs.

For a clearer comparison of training and convergence across the four architecture-matched models, Figure 6 presents the validation metric curves over 100 epochs.

As shown in Figure 6, ${\mathrm{mAP}}_{50}$, ${\mathrm{mAP}}_{75}$, and ${\mathrm{mAP}}_{50:95}$ rose rapidly during early training and gradually stabilized in the middle and late stages. Compared with YOLOv12n, YOLOv12n–LiteP2 converged to higher ${\mathrm{mAP}}_{75}$ and ${\mathrm{mAP}}_{50:95}$, reflecting better localization from the high-resolution prediction branch. DGF–YOLO maintained higher overall metrics than the baseline, supporting stronger feature representation from dynamic local-context fusion. DGF–YOLOv12n–LiteP2 remained at the top during the middle and late stages, and the separation became clearer for ${\mathrm{mAP}}_{75}$ and ${\mathrm{mAP}}_{50:95}$.

After detection accuracy and training stability were confirmed, computational efficiency was further evaluated. Parameter count, computation, inference latency, and peak memory were considered. The results are listed in Table 5.

According to Table 5, YOLOv12n contains 2.57 M parameters and requires 6.48 GFLOPs. FP32 end-to-end throughput reaches 100.60 FPS. After LiteP2 was added, the parameter count rose by only 0.15 M. However, the stride-4 high-resolution prediction grid increased computation from 6.48 to 12.29 GFLOPs, while FP32 throughput declined to 88.09 FPS. DGF–YOLOv12n–LiteP2 contains 11.68 M parameters and requires 22.89 GFLOPs. FP32 end-to-end latency reaches 19.72 ms, corresponding to 50.70 FPS. Relative to YOLOv12n, the accuracy gain came with higher parameter count, computation, and latency, with DGF accounting for most of the added complexity.

**Figure 7:**
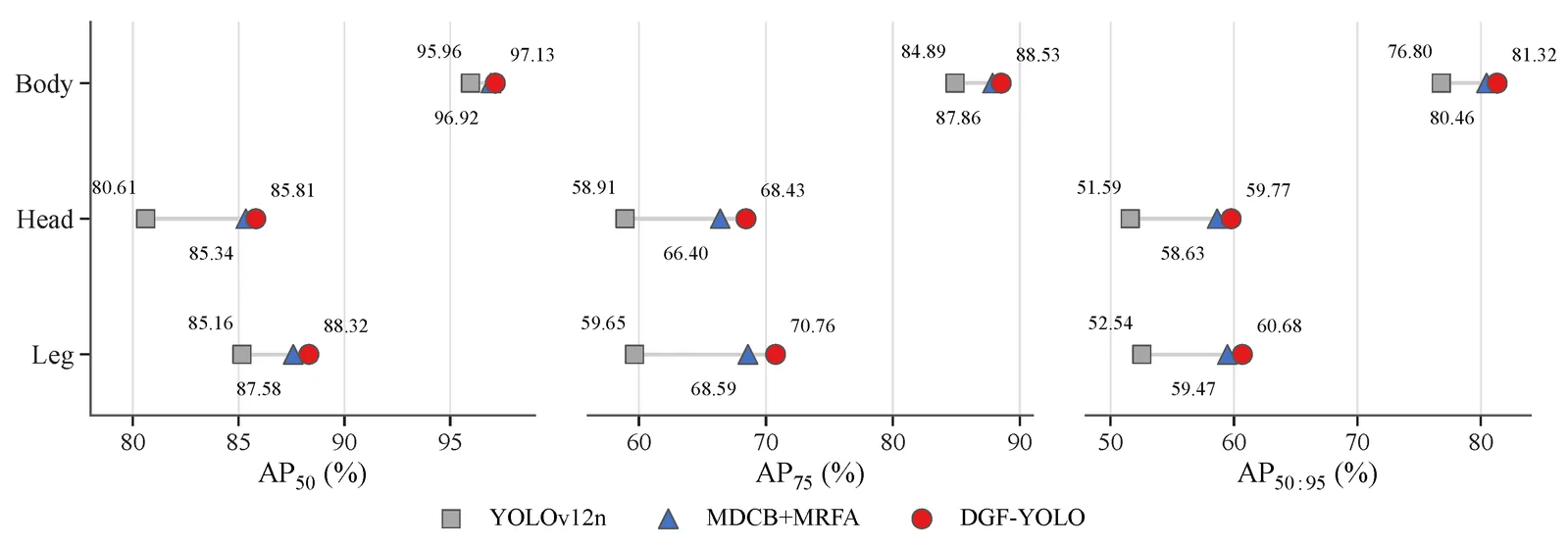
Class-wise AP comparison on the validation set.

Beyond the overall detection metrics, a class-level analysis was conducted to examine the effects of DGF on body, head, and leg. The results are provided in Figure 7.

As presented in Figure 7, DGF–YOLO improved ${\mathrm{AP}}_{50:95}$ by 4.52, 8.18, and 8.14 percentage points for body, head, and leg, respectively, relative to YOLOv12n. MDCB+MRFA produced intermediate results between YOLOv12n and DGF–YOLO. This pattern supports the complementary roles of local-detail extraction and contextual aggregation. Greater improvements were observed for head and leg categories than for body detection. DGF therefore provided greater improvement for small heads and thin legs.

Body regions generally occupy larger and more contiguous image areas, so their structural information is less vulnerable to repeated downsampling. In contrast, heads are smaller, while legs are thin, elongated, frequently occluded, and more easily confused with rail-like background structures. MDCB preserves fine local boundaries, MRFA introduces broader contextual constraints, and LiteP2 retains stride-4 spatial detail. These mechanisms therefore address the dominant failure modes of head and leg localization more directly than those of the larger body category, which helps to explain the larger gains for head and leg.

To further examine class-level stability under different training initializations, Table 6 reports held-out in-domain test results for YOLOv12n and DGF–YOLOv12n–LiteP2 across three deterministic random seeds.

According to Table 6, mean ${\mathrm{AP}}_{50:95}$ for DGF–YOLOv12n–LiteP2 increased by 4.44, 12.13, and 15.71 percentage points for body, head, and leg, respectively, relative to YOLOv12n. Larger gains were obtained for head and leg than for body. This result indicates that combining DGF and LiteP2 mainly improved small-head and thin-leg detection. Small sample standard deviations were recorded for all three classes, indicating that the class-wise gains remained relatively stable across the three deterministic random seeds.

**Figure 8:**
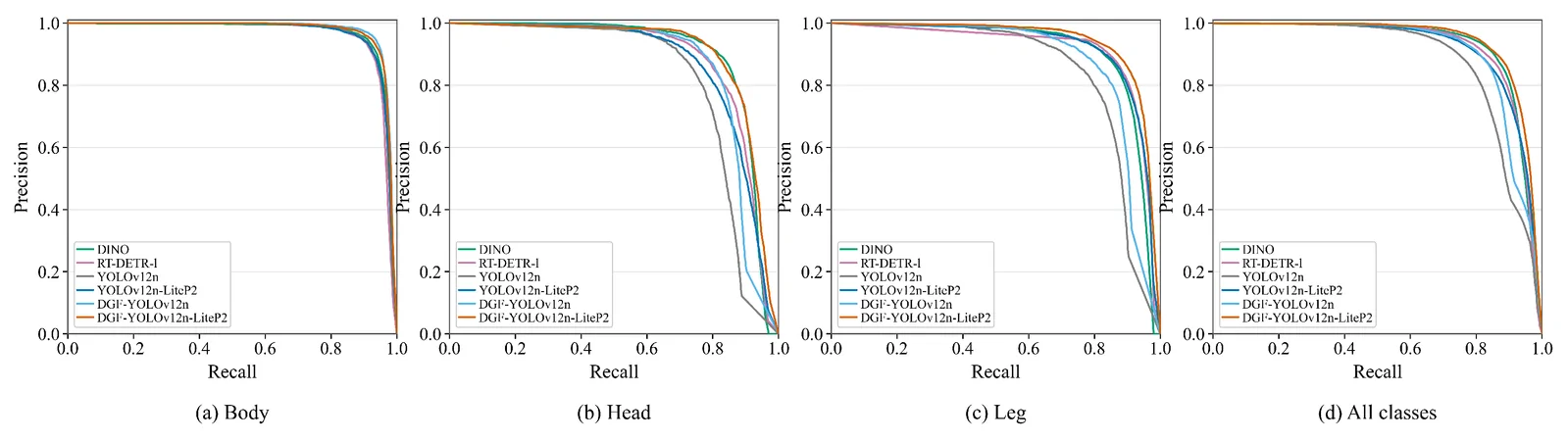
Class-wise precision–recall curves of six detectors on the held-out in-domain test set.

To compare class-wise precision and recall, Figure 8 presents the PR curves of six detection models on the held-out in-domain test set.

Figure 8 presents PR curves obtained by varying the confidence threshold. Body curves remain close, whereas wider gaps occur for the head, leg, and all classes. At high recall, DGF–YOLOv12n–LiteP2 maintained higher precision, while YOLOv12n declined earlier. For the complete model, ${\mathrm{AP}}_{50}$ reached 0.971 for body, 0.907 for head, and 0.939 for leg. A mean ${\mathrm{AP}}_{50}$ of 0.939 was obtained across the three classes.

**Figure 9:**
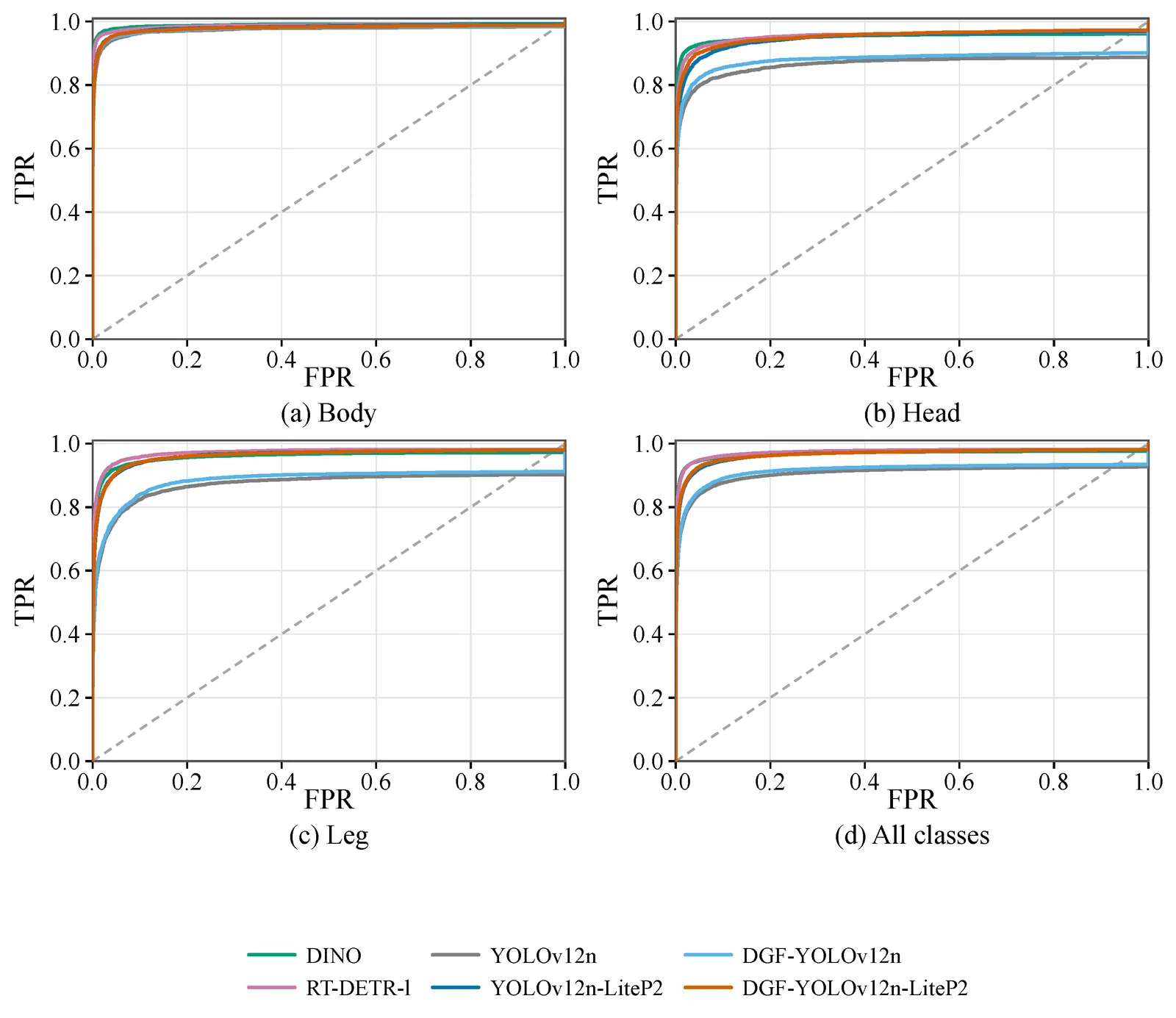
Class-wise ROC curves of six detection models on the held-out in-domain test set. The dashed diagonal denotes the random-classifier reference line.

Figure 9 presents the ROC curves of six detection models for the body, head, leg, and all classes. The horizontal and vertical axes represent the false positive rate (FPR) and true positive rate (TPR), respectively. All the models achieved high TPR values for body detection, while larger differences appeared in the head and leg categories. DGF–YOLOv12n–LiteP2 maintained a higher ROC curve position across most FPR ranges, indicating improved discrimination capability under complex detection conditions. The all-class ROC curve further demonstrates the overall advantage of the proposed model over the baseline models.

**Figure 10:**
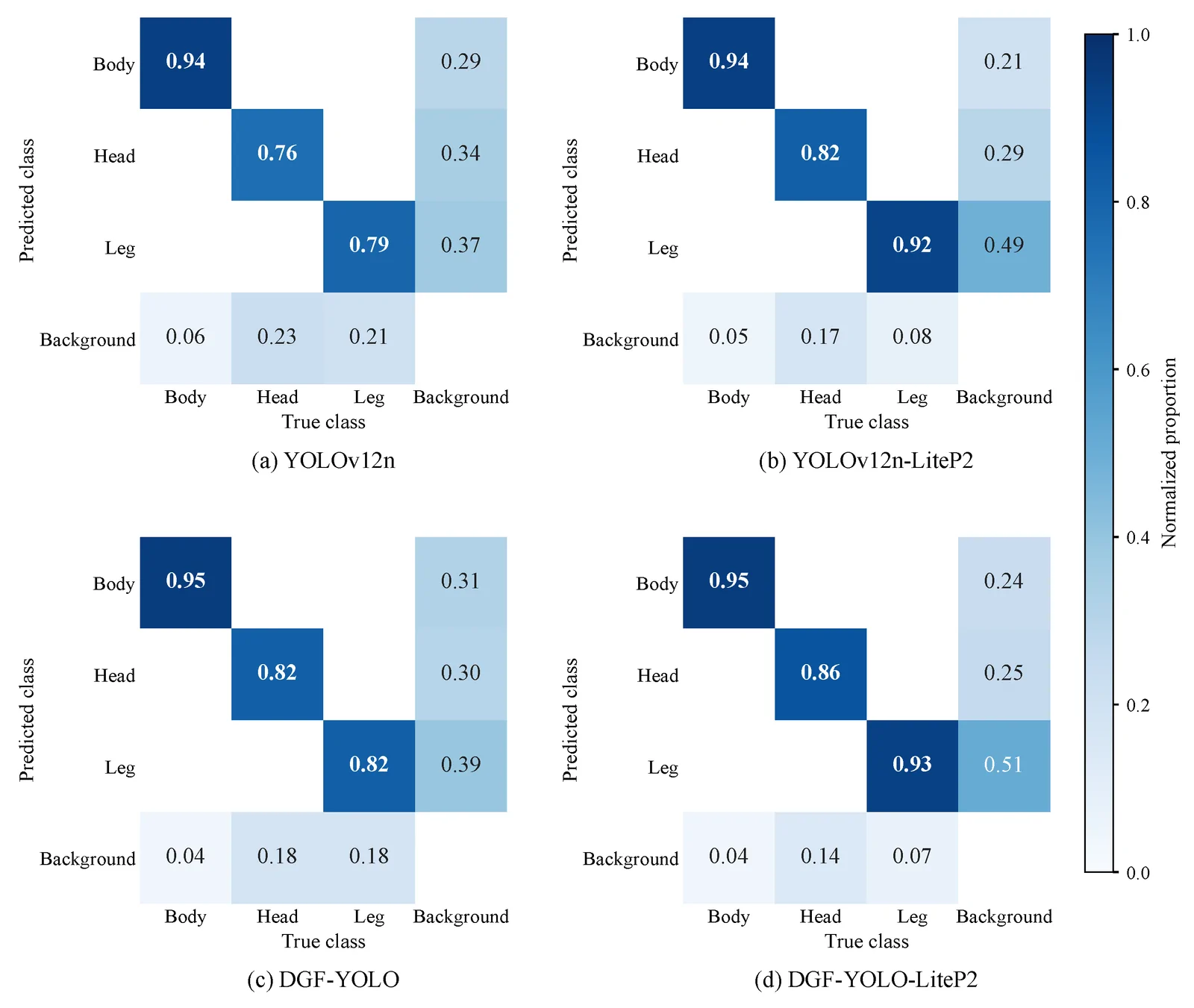
Normalized confusion matrices of four architecture-matched models on the held-out in-domain test set. Each matrix was normalized by true class.

Together with the PR curve results, the ROC analysis indicates that the proposed model improves discrimination capability, particularly for the more challenging head and leg categories. Beyond the threshold-dependent comparison, normalized confusion matrices were further examined in Figure 10. This analysis provides additional information about class recognition patterns and remaining error sources.

Figure 10 reports diagonal values of 0.95, 0.86, and 0.93 for body, head, and leg under DGF–YOLOv12n–LiteP2. All three remain above the YOLOv12n values of 0.94, 0.76, and 0.79. Most remaining errors occur between target classes and background. Some head instances remain undetected, while the leg class produces a higher proportion of background responses. Low-contrast legs, rails, and other linear structures may therefore form similar visual patterns.

The missed head instances are mainly associated with long-distance views, low illumination, and severe inter-animal occlusion, where only a small portion of the head remains visible and the available pixel information is limited. For the leg class, rails, fence bars, trough edges, shadows, overlapping limbs, and other elongated background structures can produce local patterns similar to thin cattle legs. Low contrast further reduces the distinction between the target and its surroundings. These cases indicate that DGF and LiteP2 reduce, but do not fully eliminate, ambiguity when visibility is severely degraded or when background structures closely resemble anatomical parts.

### 3.2. Ablation Studies and Module Effectiveness Validation

**Figure 11:**
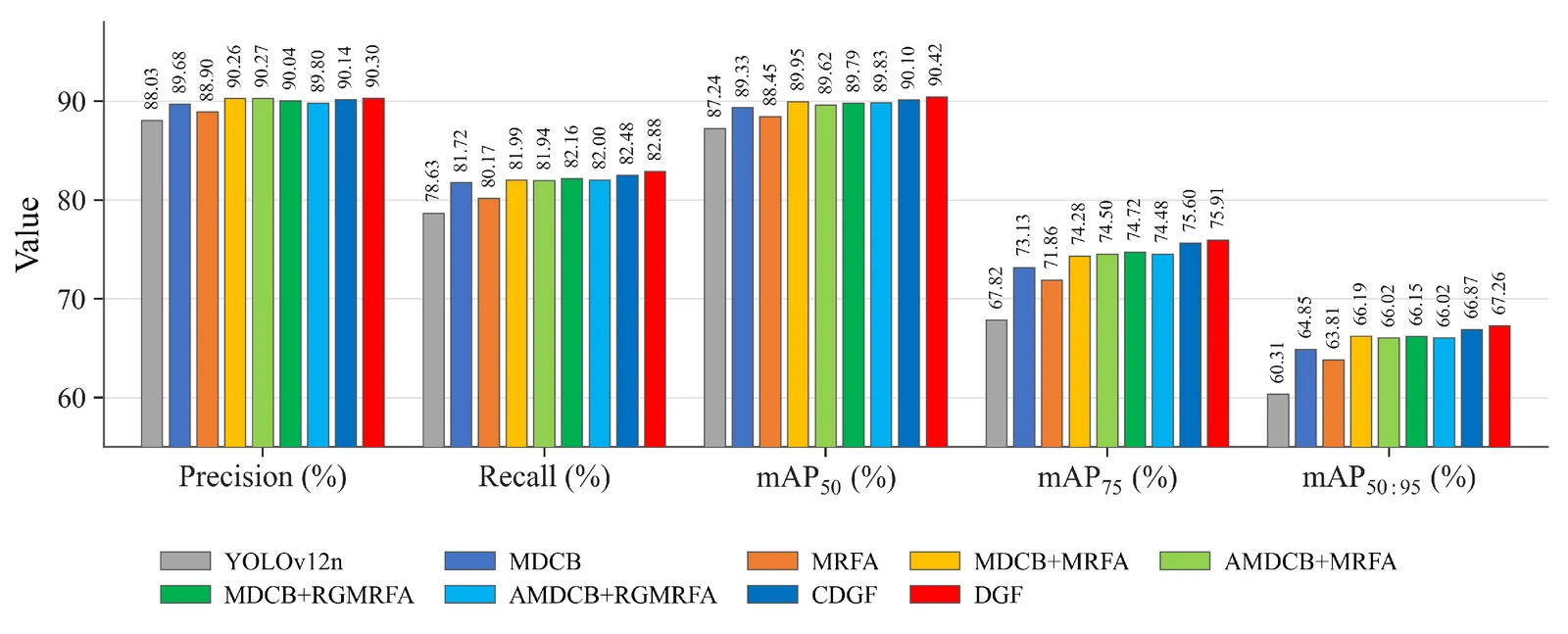
Overall validation performance of configurations E0–E8.

This subsection quantifies the synergistic gains from multi-module integration through progressive addition of the core components. The results are presented in Figure 11.

As shown in Figure 11, adding MDCB increased ${\mathrm{mAP}}_{50:95}$ from 60.31% to 64.85%. After MRFA was added to MDCB under static fusion, the metric rose further to 66.19%. This result suggests that broader contextual information complements local boundary cues.

AMDCB and RGMRFA variants in E4–E6 obtained 66.02–66.15% ${\mathrm{mAP}}_{50:95}$; these results remained below the static MDCB+MRFA configuration. This outcome indicates that more complex adaptive operations do not necessarily improve detection performance. CDGF achieved 66.87% ${\mathrm{mAP}}_{50:95}$ and 75.60% ${\mathrm{mAP}}_{75}$, while complete DGF increased these metrics to 67.26% and 75.91%. Relative to static MDCB+MRFA, DGF improved ${\mathrm{mAP}}_{50:95}$ and ${\mathrm{mAP}}_{75}$ by 1.07 and 1.63 percentage points, and the larger gain under the stricter IoU threshold corresponded to more accurate localization.

Overall, the complete framework provided better feature representation than any individual module or incomplete combination. The improvement can be attributed to the joint contribution of the DGF components. First, MDCB strengthens local boundaries and fine structures through multi-kernel paths. Second, MRFA adds contextual constraints through progressive large-receptive-field aggregation. Third, dynamic gating adjusts the contributions of the input, local, and contextual states according to the feature content. Fixed fusion weights are therefore avoided. DGF can preserve both local structure and contextual information while providing clearer localization gains under strict IoU thresholds. After integration with LiteP2, more high-resolution spatial cues remain available. Their combined effect produces the final gain of the complete model.

### 3.3. Validating the Effectiveness of LiteP2

To verify the high-resolution prediction advantage of LiteP2 for cattle body-part detection, comparative experiments were conducted in this section. LiteP2 was combined with YOLOv12n and DGF–YOLO to form YOLOv12n–LiteP2 and DGF–YOLOv12n–LiteP2. Under identical conditions, each LiteP2 model was compared with its original counterpart. The contribution of LiteP2 to detection performance was then quantified. The results are presented in Table 7.

Table 7 shows that adding LiteP2 to YOLOv12n increased recall, ${\mathrm{mAP}}_{50}$, ${\mathrm{mAP}}_{75}$, and ${\mathrm{mAP}}_{50:95}$ by 4.09, 3.82, 8.07, and 5.51 percentage points, respectively. The larger increase in ${\mathrm{mAP}}_{75}$ than in ${\mathrm{mAP}}_{50}$ indicates that stride-4 prediction improved both target detection and box localization. After LiteP2 was added to DGF–YOLO, recall rose from 82.88% to 86.34%. ${\mathrm{mAP}}_{50}$, ${\mathrm{mAP}}_{75}$, and ${\mathrm{mAP}}_{50:95}$ increased from 90.42%, 75.91%, and 67.26% to 93.71%, 80.11%, and 69.75%. The corresponding gains were 3.46, 3.29, 4.20, and 2.49 percentage points. LiteP2 therefore retained an independent contribution under dynamic fusion. Conversely, adding DGF under the fixed LiteP2 setting raised ${\mathrm{mAP}}_{50:95}$ by another 3.93 percentage points. The two structures thus provide complementary effects.

In summary, LiteP2 provided effective high-resolution prediction on the current custom dataset. Three factors explain this result. First, the stride-4 prediction grid preserves more spatial sampling points for small heads and thin legs. Second, fusion of backbone P2 features and upsampled P3 features supplies both local detail and stronger semantic information to high-resolution prediction. Third, P2 features continue to influence coarser predictions through the bottom-up path. LiteP2 therefore improves detection of small and thin targets without constructing a complete high-resolution feature pyramid. The gain still depends on the target-scale distribution.

### 3.4. Evaluation of Model Generalization

**Figure 12:**
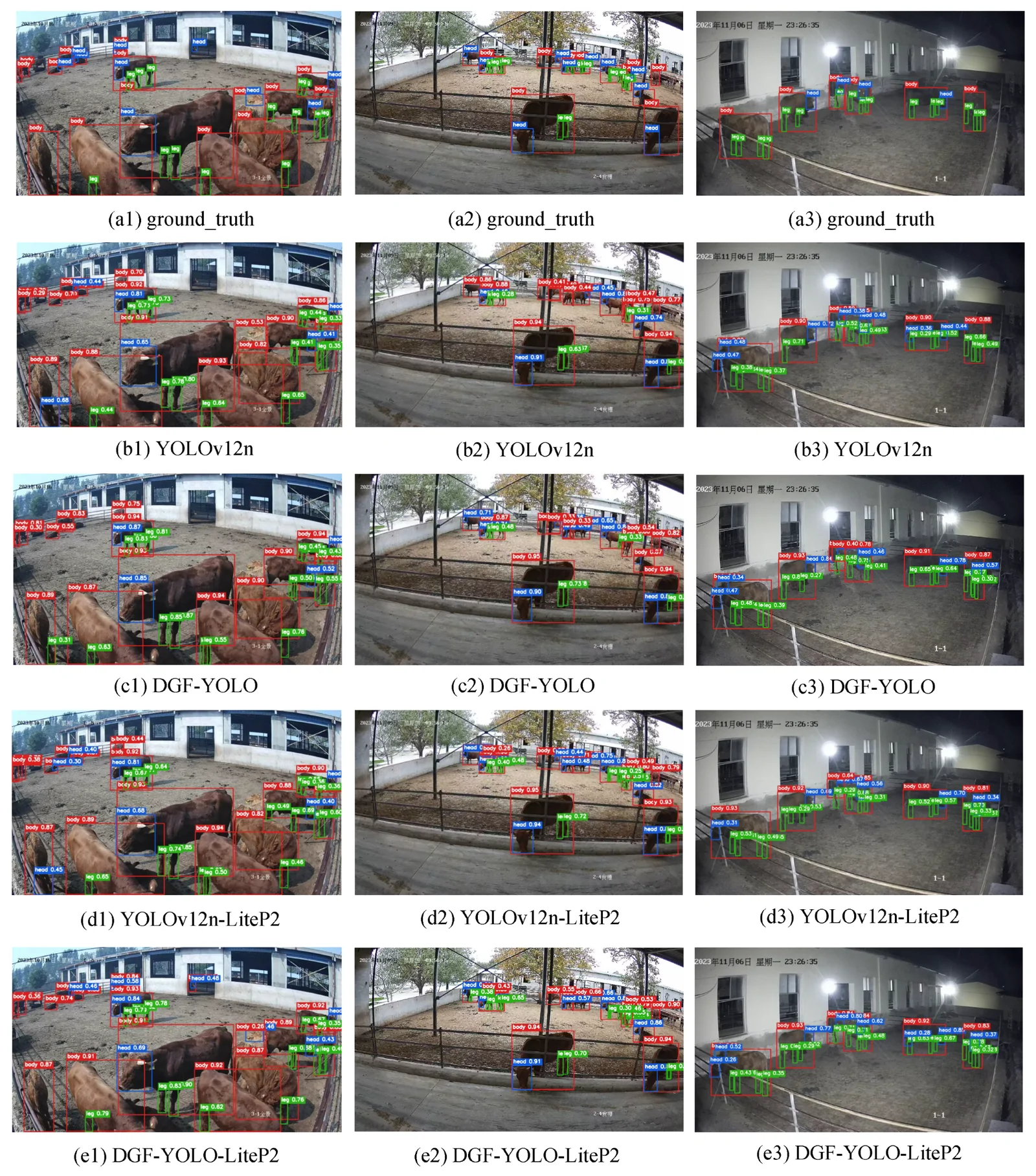
Comparison of detection results on the custom held-out in-domain test set. The Chinese characters in the timestamp overlay indicate the recording date and weekday as recorded by the surveillance camera.

To evaluate detection under complex barn conditions and examine architecture transfer, crowded and nighttime scenes were first selected from the custom held-out in-domain test set. Detection results from the architecture-matched models were compared visually. In-domain retraining and testing were then conducted on two public datasets. The results on the custom held-out in-domain test set are shown in Figure 12.

Figure 12 presents detection results in a crowded nighttime scene. Compared with YOLOv12n, the complete model reduced several missed head and leg detections. Several box positions were also improved. Repeated boxes, missed detections, and background responses still occur under dense occlusion, long distance, and low illumination.

**Figure 13:**
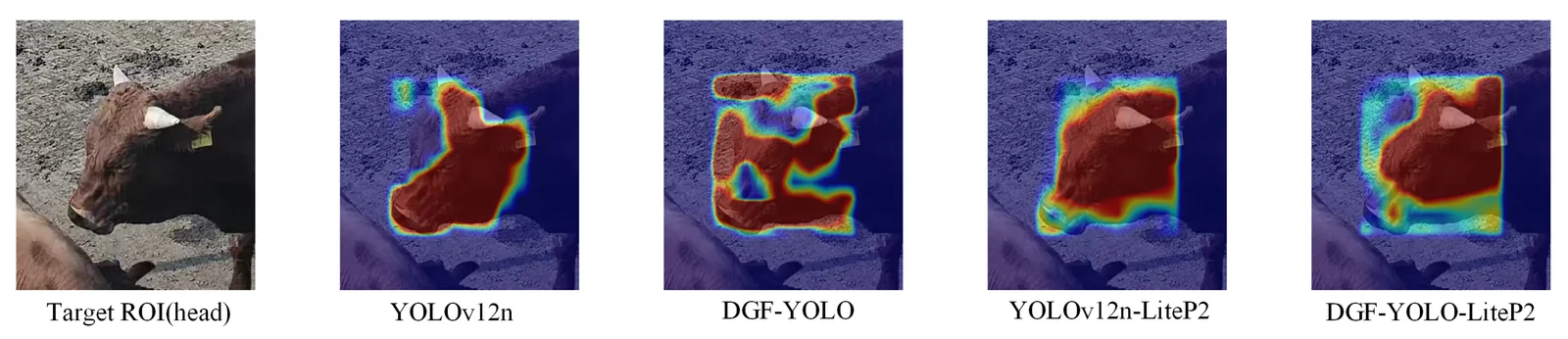
Instance-conditioned P3 response maps for a small head target.

Figure 13 presents instance-conditioned Grad-CAM responses for one small head target. Confidence scores of YOLOv12n, DGF–YOLO, YOLOv12n–LiteP2, and DGF–YOLOv12n–LiteP2 reached 0.653, 0.854, 0.676, and 0.695, respectively. The corresponding matched-box IoU values were 0.950, 0.945, 0.921, and 0.932.

To further evaluate architecture transfer, supplementary experiments were conducted on two public datasets. Every model was retrained on the training split of the corresponding dataset. Evaluation was completed on the held-out test set. The results are reported in Table 8.

According to Table 8, DGF–YOLOv12n–LiteP2 reached 69.39% ${\mathrm{mAP}}_{50:95}$ on the public cattle body-part benchmark. This value was 1.21 percentage points above the YOLOv12n result. DGF–YOLO recorded the highest value of 69.91%, which was 0.52 percentage points above the complete-model result. This result indicates that stride-4 prediction may provide no additional gain when targets are relatively large and fully visible. On Public-Cow-100, the complete model increased ${\mathrm{mAP}}_{50:95}$ from 56.60% to 58.50%. Precision, recall, ${\mathrm{mAP}}_{50}$, and ${\mathrm{mAP}}_{75}$ also increased.

**Figure 14:**
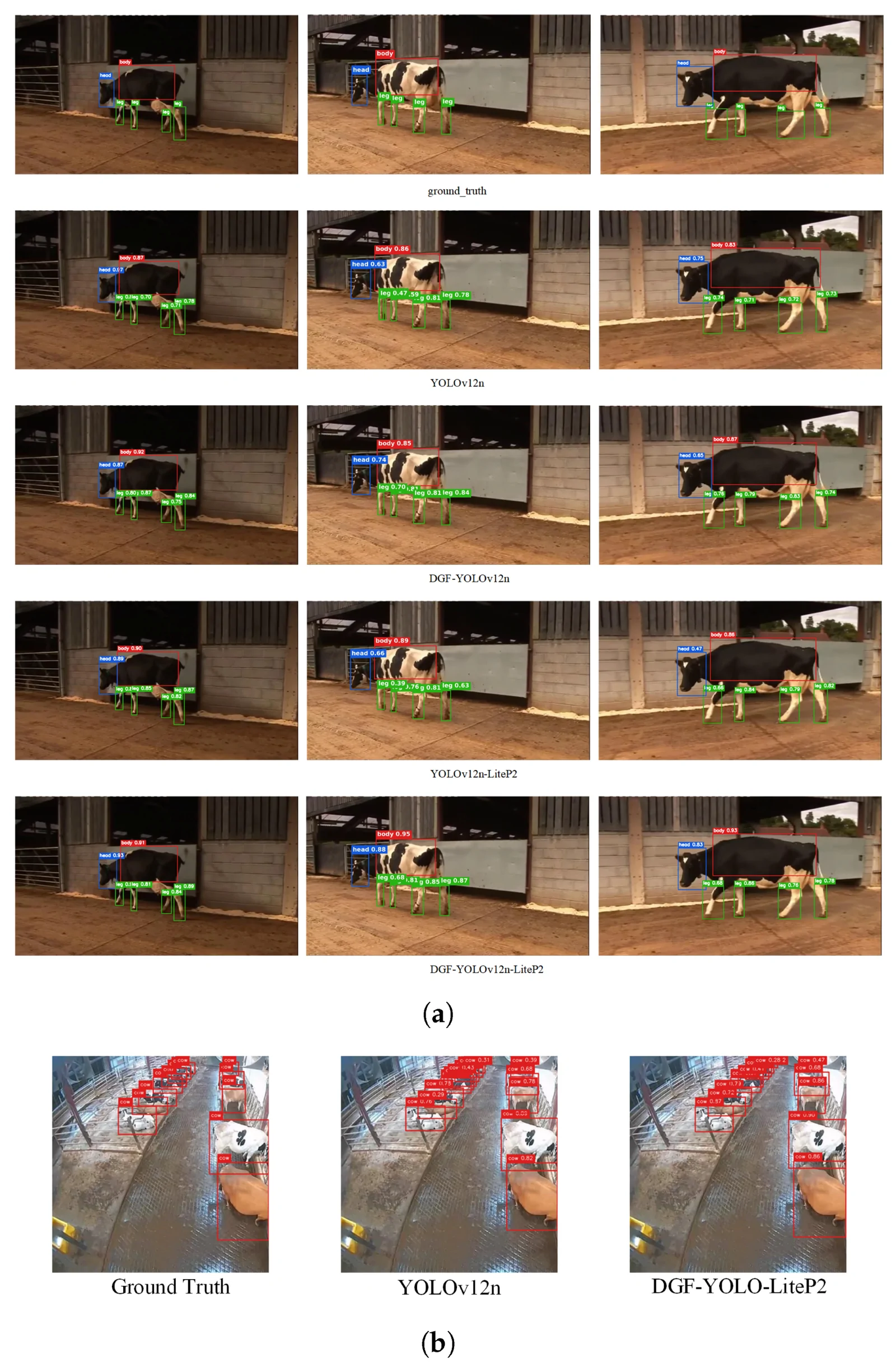
Qualitative architecture-transfer examples. Each panel presents predictions from models retrained on the corresponding public dataset, (**a**) Public cattle body-part benchmark: ground truth, YOLOv12n, DGF–YOLO, YOLOv12n–LiteP2, and DGF–YOLOv12n–LiteP2, (**b**) Public whole-cow benchmark: ground truth, YOLOv12n, and DGF–YOLOv12n–LiteP2.

To further evaluate detection performance on a public cattle body-part dataset, qualitative comparisons were conducted on the Cattle Body Parts Dataset for Object Detection. The dataset contains 428 images. Figure 14a presents the ground-truth annotations and the predictions from YOLOv12n, DGF–YOLO, YOLOv12n–LiteP2, and DGF–YOLOv12n–LiteP2. All four models locate the main body parts under scale variation, partial occlusion, and overlapping regions. Compared with YOLOv12n, DGF–YOLOv12n–LiteP2 produces more complete predictions for several head and leg regions. Its prediction boxes also show closer alignment with the annotations.

Whole-cow detection was further evaluated on the COLO dataset, which contains 1254 images. Figure 14b compares the ground-truth annotations and the predictions from YOLOv12n and DGF–YOLOv12n–LiteP2. DGF–YOLOv12n–LiteP2 provides more complete target localization and closer correspondence with the annotated cattle regions.

**Figure 15:**
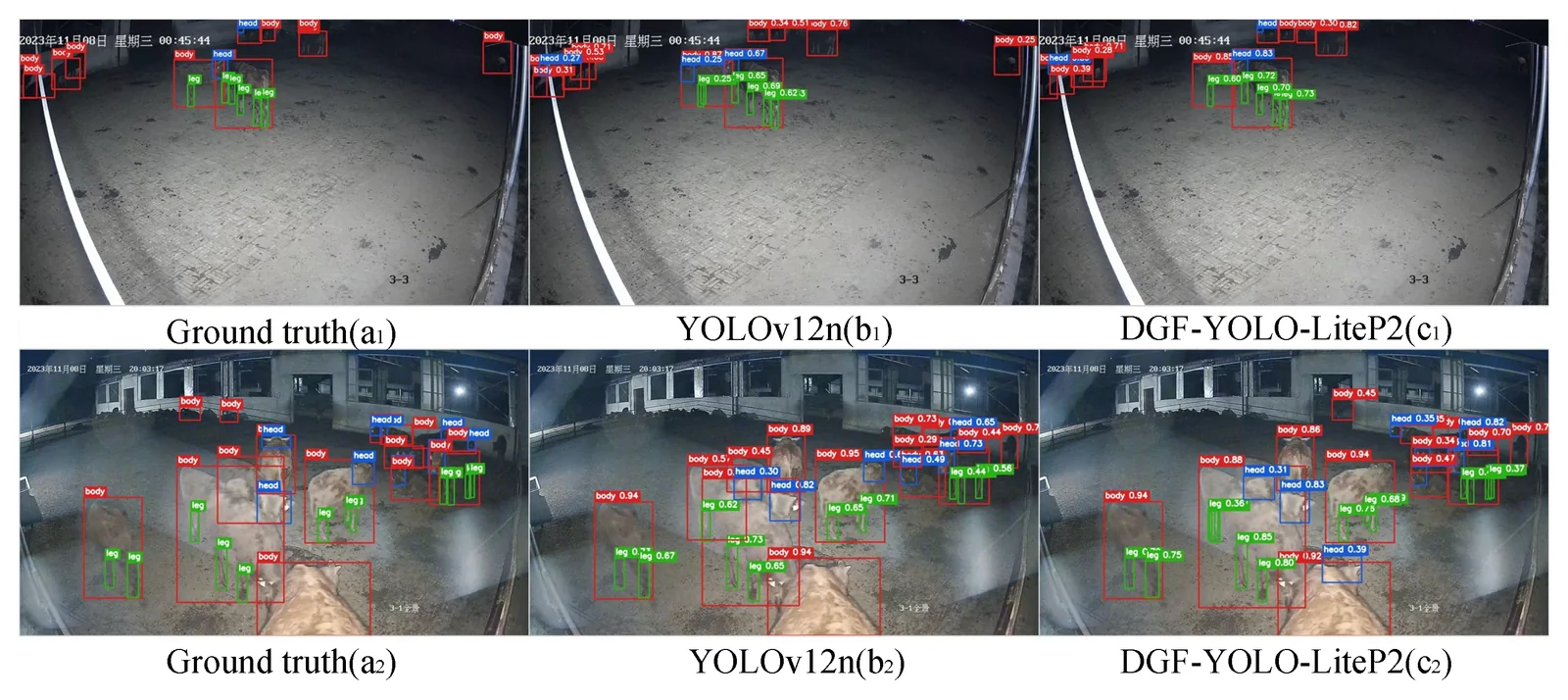
Representative failure cases, including long-distance low-light scenes, dense occlusion, and rail interference. The Chinese characters in the timestamp overlay indicate the recording date and weekday as recorded by the surveillance camera.

Figure 15 visually confirms the remaining failure modes under long-distance, low-light, densely occluded, and rail-interference conditions. Missed distant parts, duplicate boxes near overlapping animals, and false leg responses caused by linear background structures are still observed, although the complete model reduces some of these errors.

Overall, the proposed DGF–YOLOv12n–LiteP2 produced favorable cattle body-part detection results in complex barns. The improvement can be attributed to the cooperation between dynamic local-context fusion and high-resolution prediction. First, MDCB preserves local boundaries and fine structures through multi-kernel paths. Second, MRFA adds broader contextual information through progressive large-receptive-field aggregation. Dynamic gating then adjusts the contributions of local and contextual representations according to the input content. Third, LiteP2 preserves stride-4 spatial information for direct prediction and subsequent bottom-up reconstruction. The complete model consequently provides larger gains for small heads and thin legs. Training stability and architecture transfer were maintained. The accuracy improvement was accompanied by increased computational complexity and reduced inference throughput. Low illumination, occlusion, and rail interference remain open problems.

## 4. Discussion

This study presents DGF–YOLOv12n–LiteP2 for cattle body-part detection in complex barn environments. Two main challenges receive attention. The first concerns the insufficient balance between local boundary details and broader contextual information. The second concerns spatial information loss after repeated downsampling. On the held-out in-domain test set, DGF–YOLOv12n–LiteP2 achieved 93.92% ${\mathrm{mAP}}_{50}$, 79.27% ${\mathrm{mAP}}_{75}$, and 69.88% ${\mathrm{mAP}}_{50:95}$. Compared with YOLOv12n, the proposed model increased ${\mathrm{mAP}}_{50:95}$ by 9.61 percentage points. Across three deterministic training seeds, the mean ${\mathrm{mAP}}_{50:95}$ reached 70.03±0.30%. These results support the complementary roles of dynamic local and contextual fusion and high-resolution prediction.

The main contribution of this study lies in combining two complementary mechanisms. DGF performs input-conditioned local–context feature selection, whereas LiteP2 preserves stride-4 spatial information for small and slender body parts. The structured ablation, multi-seed evaluation, public-dataset transfer analysis, and efficiency measurements further distinguish the effects and trade-offs of these two components.

At the feature-representation level, local boundaries and structural details are preserved through the parallel multi-kernel paths in MDCB. Broader contextual information is introduced by MRFA through progressive large-kernel aggregation. Among the individual modules, MDCB produced the largest improvement and increased ${\mathrm{mAP}}_{50:95}$ from 60.31% to 64.85%. MRFA reached 63.81%, while the static combination further raised this value to 66.19%. After static fusion was replaced by DGF, ${\mathrm{mAP}}_{50:95}$ increased to 67.26%. A larger improvement also appeared at the stricter IoU threshold. This pattern suggests that adaptive selection between local and contextual representations contributes to more accurate localization. However, the current ablation results provide indirect support for this explanation. A direct causal relationship between gate behavior and performance cannot be confirmed.

Spatial information for small and slender body parts often decreases after repeated downsampling. To reduce this loss, LiteP2 introduces stride-4 prediction. After LiteP2 was added to YOLOv12n, recall increased from 78.63% to 82.72%, while ${\mathrm{mAP}}_{50:95}$ increased from 60.31% to 65.82%. With LiteP2 retained, the addition of DGF further raised recall to 86.34% and ${\mathrm{mAP}}_{50:95}$ to 69.75%. The class-wise results showed larger improvements for the head and leg categories than for the body category. Across three seeds, the AP_50:95_ improvements over YOLOv12n reached 4.44 percentage points for body, 12.13 points for head, and 15.71 points for leg. This pattern agrees with the smaller spatial extent, thin structure, and frequent occlusion of head and leg instances.

This class-wise pattern is qualitatively consistent with earlier cattle-part detectors that improved scale-sensitive representation through filtering, adaptive spatial feature fusion, or attention [9,10,11]. The present results extend those observations by separating two mechanisms: DGF performs input-conditioned selection between local boundary detail and broader contextual evidence within each detection level, whereas LiteP2 retains a stride-4 prediction path. The remaining errors under low illumination, dense occlusion, and linear background interference also agree with the broader challenges addressed by low-illumination enhancement and density-aware perception methods [32,33]. These comparisons are qualitative because the published studies use different datasets, splits, target definitions, and evaluation settings; direct numerical ranking would therefore be misleading.

The accuracy improvement came with higher computational cost. DGF–YOLOv12n–LiteP2 contained 11.68 M parameters, required 22.89 GFLOPs, and achieved 50.70 FPS during the batch-one FP32 benchmark on an RTX 4090. In comparison, YOLOv12n contained 2.57 M parameters, required 6.48 GFLOPs, and achieved 100.60 FPS. The term “Lite” describes the compact structure of the additional P2 branch rather than the complete detector. Real-time throughput was maintained on a high-performance desktop GPU. However, performance on embedded agricultural hardware still requires further evaluation.

After in-domain retraining, the public-dataset experiments provided additional evidence of architecture transfer. On one cattle body-part benchmark, DGF–YOLO recorded a slightly higher value than the complete model. This result indicates that high-resolution prediction may not benefit every target-scale distribution. Therefore, the current evidence supports architecture transfer rather than zero-shot cross-farm generalization.

Several limitations remain. The primary dataset came from one farm and covered a limited collection period. Detailed metadata on camera installation and image acquisition, together with verified animal-level demographic information, were not retained with the analytical dataset, which limits acquisition-level reproducibility and demographic subgroup analysis. Downstream behavior recognition, gait analysis, and health-monitoring tasks were not evaluated. Future research should include multi-farm and multi-season validation, more training seeds, multimodal sensing, downstream livestock-monitoring experiments, and deployment tests on agricultural edge devices.

## 5. Conclusions

This study presents DGF–YOLOv12n–LiteP2 for cattle body-part detection in complex barn environments. The proposed framework addresses two different detection bottlenecks. DGF improves feature representation through MDCB-based local-detail extraction, MRFA-based contextual aggregation, triplet-conditioned feature selection, and bounded spatial refinement. LiteP2 reuses the backbone P2 feature and introduces stride-4 prediction to preserve spatial information for small heads and thin legs without constructing a complete high-resolution feature pyramid.

The paired architecture comparisons indicate that DGF and LiteP2 provide complementary contributions. DGF strengthens local–context feature representation, whereas LiteP2 improves high-resolution spatial sampling and bottom-up feature reconstruction. On the held-out in-domain test set, DGF–YOLOv12n–LiteP2 achieved 93.92% ${\mathrm{mAP}}_{50}$, 79.27% ${\mathrm{mAP}}_{75}$, and 69.88% ${\mathrm{mAP}}_{50:95}$. Compared with YOLOv12n, the ${\mathrm{mAP}}_{50:95}$ increased by 9.61 percentage points. Across three deterministic training seeds, the mean ${\mathrm{mAP}}_{50:95}$ reached 70.03±0.30%. Larger class-wise improvements were observed for the smaller and thinner head and leg categories, supporting the combined roles of dynamic local–context fusion and high-resolution prediction under the evaluated barn conditions.

The complete model contains 11.68 M parameters, requires 22.89 GFLOPs, and reaches 50.70 FPS in the batch-one FP32 benchmark on an RTX 4090. DGF accounts for most of the additional parameters, while the high-resolution prediction branch also increases computation. Therefore, the accuracy improvement is accompanied by higher computational cost and inference latency than YOLOv12n. The current evidence is limited to the evaluated datasets and hardware platform. Future research will consider different farms, seasons, camera configurations, downstream livestock-monitoring tasks, and agricultural edge devices.

## Figures and Tables

**Table 1:** Dataset composition and annotated instances.

Split	Raw	Valid	Body	Head	Leg
Train	4896	4894	32,440	21,844	37,748
Validation	1399	1398	9430	6390	11,028
Test	699	699	4562	3067	5793
Total	6994	6991	46,432	31,301	54,569

**Table 2:** Overall performance comparison on the held-out validation set.

Model	P	R	${\mathrm{mAP}}_{50}$	${\mathrm{mAP}}_{75}$	${\mathrm{mAP}}_{50:95}$
Faster R-CNN R50-FPN	–	–	74.70	63.50	55.00
DINO R50	–	–	92.50	74.70	66.50
RT-DETR-l	89.98	85.59	92.71	77.69	68.86
YOLOv5n	89.03	79.32	87.92	69.78	62.15
YOLOv8n	89.69	80.72	88.78	71.61	63.54
YOLO11n	89.01	78.95	87.80	70.60	62.73
YOLO11s	91.16	84.24	91.70	78.97	69.64
YOLOv12n	88.03	78.63	87.24	67.82	60.31
DGF–YOLO	90.30	82.88	90.42	75.91	67.26
YOLOv12n–LiteP2	88.63	82.72	91.06	75.89	65.82
DGF–YOLOv12n–LiteP2	91.24	86.34	93.71	80.11	69.75

**Table 3:** Performance comparison of selected models on the held-out in-domain test set.

Model	P	R	${\mathrm{mAP}}_{50}$	${\mathrm{mAP}}_{75}$	${\mathrm{mAP}}_{50:95}$
YOLO11s	90.98	84.89	91.75	78.58	69.67
YOLOv12n	88.42	79.55	87.64	66.76	60.27
DGF–YOLO	90.00	83.59	90.60	75.21	67.06
YOLOv12n–LiteP2	89.49	83.99	91.86	75.30	66.08
RT-DETR-l	90.20	84.53	92.24	78.04	68.81
DINO R50	–	–	92.21	74.56	66.09
DGF–YOLOv12n–LiteP2	91.47	86.73	93.92	79.27	69.88

**Table 4:** Held-out in-domain test results across three deterministic random seeds.

Model	P	R	${\mathrm{mAP}}_{50}$	${\mathrm{mAP}}_{75}$	${\mathrm{mAP}}_{50:95}$	95% CI
YOLOv12n	87.26±0.17	79.53±0.63	87.12±0.15	65.27±0.47	59.27±0.47	[58.09, 60.44]
DGF–YOLO	90.16±0.40	83.29±0.23	90.38±0.13	74.27±0.34	66.36±0.32	[65.57, 67.14]
YOLOv12n–LiteP2	89.36±0.58	84.29±0.09	92.05±0.27	73.95±1.34	65.12±0.85	[63.00, 67.25]
DGF–YOLOv12n–LiteP2	91.46±0.03	87.08±0.40	93.98±0.17	79.78±0.54	70.03±0.30	[69.28, 70.78]

**Table 5:** Unified batch-one efficiency comparison on an RTX 4090.

Model	Params (M)	GFLOPs	FP32 E2E (ms)	FP32 FPS	FP16 E2E (ms)	FP16 FPS	FP32 Peak (MB)
YOLOv12n (baseline)	2.57	6.48	9.94	100.60	10.19	98.15	182.27
+DGF–YOLO	11.52	22.45	17.81	56.16	18.76	53.32	236.05
+YOLOv12n–LiteP2	2.72	12.29	11.35	88.09	11.72	85.29	199.13
+DGF–YOLOv12n–LiteP2	11.68	22.89	19.72	50.70	20.08	49.79	240.87

**Table 6:** Three-seed class-wise test AP_50:95_.

Model	Body	Head	Leg
YOLOv12n	76.53±0.44	51.22±0.50	50.06±0.64
DGF–YOLOv12n–LiteP2	80.97±0.13	63.35±0.36	65.77±0.54

**Table 7:** Architecture-matched validation results for the paired evaluation of DGF and LiteP2.

Model	P	R	${\mathrm{mAP}}_{50}$	${\mathrm{mAP}}_{75}$	${\mathrm{mAP}}_{50:95}$
YOLOv12n	88.03	78.63	87.24	67.82	60.31
DGF–YOLO	90.30	82.88	90.42	75.91	67.26
YOLOv12n–LiteP2	88.63 (+0.60)	82.72 (+4.09)	91.06 (+3.82)	75.89 (+8.07)	65.82 (+5.51)
DGF–YOLOv12n–LiteP2	91.24 (+2.61)	86.34 (+3.62)	93.71 (+2.65)	80.11 (+4.22)	69.75 (+3.93)

Note:Values in parentheses for YOLOv12n–LiteP2 indicate changes relative to YOLOv12n. Values in parentheses for DGF–YOLOv12n–LiteP2 indicate DGF gains over YOLOv12n–LiteP2 under the fixed LiteP2 setting.

**Table 8:** Held-out in-domain test results after in-domain retraining on public datasets.

Dataset	Model	P	R	${\mathrm{mAP}}_{50}$	${\mathrm{mAP}}_{75}$	${\mathrm{mAP}}_{50:95}$
Cattle body parts	YOLOv12n	98.75	99.79	99.46	82.55	68.18
	DGF–YOLO	99.04	99.35	99.31	83.09	69.91
	YOLOv12n–LiteP2	98.83	98.54	99.06	72.25	65.96
	DGF–YOLOv12n–LiteP2	98.71	99.79	99.44	81.13	69.39
Public-Cow-100	YOLOv12n	88.80	82.14	91.33	61.75	56.60
	DGF–YOLOv12n–LiteP2	91.28	83.00	92.39	63.37	58.50

## Data Availability

The custom cattle body-part dataset is not publicly available because access is subject to institutional and farm approval. The public benchmark datasets used in the transfer experiments are available from the sources cited in the manuscript. Inquiries concerning the custom dataset may be directed to the corresponding author and will be considered subject to the applicable institutional and farm-access conditions.
